# THE LANDSCAPE AND OUTLOOK FOR CONTINUED WORK LATER IN LIFE IN THE 2020S

**DOI:** 10.1093/geroni/igad104.1147

**Published:** 2023-12-21

**Authors:** Kevin Cahill, Benjamin Shaw, Brian Beach

## Abstract

For the first time in more than three decades, older workers’ labor force participation rates are no longer increasing. Even more remarkable is that this trend contrasts with changes among younger workers, who have experienced increases in labor force participation in the wake of the Covid-19 pandemic. A narrative that has existed for more than two decades—that older workers have been the exception to reductions in labor force participation generally—may be coming to an end. A question then is, in the 2020s, will older workers who exited the labor force during the pandemic return? This symposium explores the new landscape that older workers face. The first paper addresses work-related trade-offs and the extent to which older workers assess the benefits and costs of continued work later in life. The second paper explores how pension eligibility triggers a new stage of the life course, in which older workers can leverage their newfound financial gains to improve work conditions or, alternatively, to exit the labor force. The third paper explores, specifically, how the Covid pandemic changed the workplace for older workers. The fourth paper rounds out the symposium by exploring the relationship between income and health in recent years in both the United States and in China. The flexibility of the U.S. labor force enables fairly abrupt shifts in workforce dynamics in response to macroeconomic changes and other events. The Covid-19 pandemic was one such shift. This symposium provides insight into the post-pandemic labor market for older workers. This is an Economics of Aging Interest Group Sponsored Symposium.

